# Nanocrystal Agglomerates of Curcumin Prepared by Electrospray Drying as an Excipient-Free Dry Powder for Inhalation

**DOI:** 10.1155/2024/6288621

**Published:** 2024-09-06

**Authors:** Shahram Emami, Zahra Hemmati, Shadi Yaqoubi, Hamed Hamishehkar, Amin Alvani

**Affiliations:** ^1^ Department of Pharmaceutics School of Pharmacy Urmia University of Medical Sciences, Urmia, Iran; ^2^ Student Research Committee School of Pharmacy Urmia University of Medical Sciences, Urmia, Iran; ^3^ Drug Applied Research Center Tabriz University of Medical Sciences, Tabriz, Iran; ^4^ Student Research Committee Faculty of Pharmacy Tabriz University of Medical Sciences, Tabriz, Iran

## Abstract

Curcumin has shown beneficial effects on pulmonary diseases with chronic inflammation or abnormal inflammatory responses, including chronic obstructive pulmonary disease, asthma, and pulmonary fibrosis. Clinical applications of curcumin are limited due to its chemical instability in solution, low water solubility, poor oral bioavailability, and intestinal and liver first-pass metabolism. Pulmonary delivery of curcumin can address these challenges and provide a high concentration in lung tissues. The purpose of the current work was to prepare a novel inhalable dry powder of curcumin nanocrystals without added excipients using electrospray drying (ED) with improved dissolution and aerosolization properties. ED of curcumin was performed at 2 and 4% w/v concentrations in acetone. Physicochemical properties of the formulated powders were evaluated by powder X-ray diffraction (PXRD), Fourier transform infrared spectroscopy (FTIR), scanning electron microscopy (SEM), density and powder flow measurements, and in vitro dissolution. The in vitro deposition studies were conducted using next-generation impactor (NGI) and aerosol performance and aerodynamic particle size parameters were calculated for prepared formulations. ED could produce agglomerates of nanocrystals with a size of about 500 nm at an acceptable yield of about 50%. PXRD and FTIR data revealed that prepared nanocrystals were in a stable crystalline state. The bulk and tapped density of prepared agglomerates were in the range appropriate for pulmonary delivery. Formed nanocrystals could significantly improve the dissolution rate of water-insoluble curcumin. The optimized formulation exhibited acceptable recovered dose percentage, high emitted dose percentage, optimum mean mass median aerodynamic diameter, small geometric standard deviation, and high fine-particle fraction that favors delivery of curcumin to the deep lung regions. The ED proved to be an efficient technique to prepare curcumin nanocrystals for pulmonary delivery in a single step, at a mild condition, and with no surfactant.

## 1. Introduction

Curcumin is a bioactive compound derived from turmeric rhizomes [1]. This polyphenolic compound exhibits potent anti-inflammatory, antioxidant, antifibrotic, and antimicrobial effects, which introduce it as an attractive option to treat a variety of diseases, including pulmonary diseases [2]. Recent studies have showed the positive effects of curcumin on pulmonary diseases with chronic inflammation or abnormal inflammatory responses, such as chronic obstructive pulmonary disease [3], asthma [4], and pulmonary fibrosis [5]. Along with the mentioned pharmacological effects, curcumin also exerts antitumor activity against lung cancer [6].

Despite promising pharmacological effects, clinical applications of curcumin are limited due to its chemical instability in solution, low water solubility, and poor oral bioavailability [7]. Furthermore, studies have shown that curcumin undergoes rapid metabolism and elimination in the gastrointestinal tract and liver, leading to low plasma concentrations and limited therapeutic efficacy [8]. Therefore, high oral doses and repeated dosing are needed to elicit desirable therapeutic effects.

Direct administration of drugs to the pulmonary region is a feasible and effective option for managing pulmonary disorders including cystic fibrosis, asthma, chronic pulmonary infections, or lung cancer [9]. This local delivery not only provides high drug concentrations in lung tissues with lower doses but also avoids systemic side effects [10]. Furthermore, pulmonary delivery has rapid onset of action and can eliminate intestinal and liver first-pass metabolism [11]. For example, Hu et al. produced a powder of curcumin using wet milling combined with the spray drying and compared its plasma and lung concentrations with orally administered curcumin in rabbits [12]. Developed powder showed six times more rapid absorption and about 3.2-fold higher bioavailability than oral form.

Drugs are locally delivered to different pulmonary regions by specifically designed devices called inhalers. There are four types of inhalers including nebulizers, pressurized metered dose inhalers, soft mist inhalers, and dry powder inhalers (DPIs) [13]. DPIs offer several advantages over other inhalers, including no need for propellants, portable and convenient, high drug loading capacity, accurate dosing, prolonged action, and higher physical and chemical stability. To develop a robust, stable, and reproducible DPI product, usually functional excipients such as carriers are used by formulators [14]. However, only a very limited number of excipients have been approved by regulatory authorities for pulmonary drug delivery [15]. Development of excipient-free or “drug-only” DPIs eliminate possible excipient-related toxicity, reduces the time and cost needed for toxicity assessments, and enhances the chance of approval by regulatory authorities. Drug-only DPIs contain weak and redispersible agglomerates of micronized particles with size smaller than 5 *μ*m [16].

Different particle engineering methods such as spray drying [17], spray freeze drying [18], mechanical milling [19], thin film freezing [20], and electrospray drying [21] have been used to formulate DPIs with optimum size and good flowability to target deep regions of pulmonary tract efficiently. Electrohydrodynamic atomization or electrospray drying (ED) uses an electric field to produce very fine-charged droplets from a solution or suspension of formulation components in a volatile solvent [22]. Rapid evaporation of the solvent from formed fine liquid aerosol results in the formation of dry powder. ED is a simple, one-step method that does not require adding surfactants and stabilizers and is carried out under ambient temperature/pressure conditions [23]. Another advantage of ED is that it can produce dry powders with very low solvent residual in a short time in contrast to other drying methods, such as freeze drying [24]. Furthermore, by adjusting the process parameters, it is possible to produce particles with controlled crystallinity, narrow size distribution, and desired morphology. The main operating parameters of ED are flow rate, feed concentration, applied voltage, nozzle diameter, and working distance [25]. This method has been used to produce cocrystals [26], nanocrystals [27], and amorphous solid dispersions [28]. From industrial point of view, availability of industrial scale ED equipment such as Fluidnatek (BioInicia, Spain) makes this technology accessible for real world applications [25].

Recent studies have demonstrated the potential of ED for producing various types of inhalable particles for pulmonary drug delivery [29, 30]. This method can produce inhalable polymeric nanoparticles and microparticles with high drug loading and encapsulation efficiency [31]. Pure drug nanocrystals are another type of inhalable particles that can be produced by ED. The advantages of using nanocrystals in formulating of DPIs are high drug loading, improved dissolution, and reduced pulmonary clearance [32]. Until now, there are some published works about using ED to develop nanocrystals intended for oral delivery [27, 33]. However, very few is known about the potential use of ED for formulating pure drug nanocrystals in the form of DPIs for pulmonary delivery.

As far as we know, the current study is the first study that uses ED to formulate pure drug nanocrystals of a poorly water-soluble compound in the form of DPI and evaluate their potential use for pulmonary delivery. Considering the advantages of particle engineering by ED, the usefulness of pulmonary delivery of curcumin, and significance of dissolution in pulmonary absorption of water-insoluble curcumin, the purpose of the current study was to prepare excipient-free inhalable nanocrystal agglomerates of curcumin particles by ED with improved dissolution and aerosolization properties. Physicochemical properties of the formulated powders were evaluated by powder X-ray diffraction (PXRD), Fourier transform infrared spectroscopy (FTIR), scanning electron microscopy (SEM), density and powder flow measurements, and in vitro dissolution. The in vitro deposition studies were conducted by next-generation impactor (NGI) and aerosol performance and aerodynamic particle size parameters were calculated for prepared formulations.

## 2. Materials and Methods

### 2.1. Materials

Raw curcumin (RCUR), Tween 80, and sodium lauryl sulfate (SLS) were supplied by Merck (Darmstadt, Germany). Ethanol and acetone were obtained from Dr. Mojallali Chemical Industries Complex Co (Tehran, Iran). All other reagents employed were of analytical reagent grade.

### 2.2. Electrospray Drying (ED)

**Figure 1:**
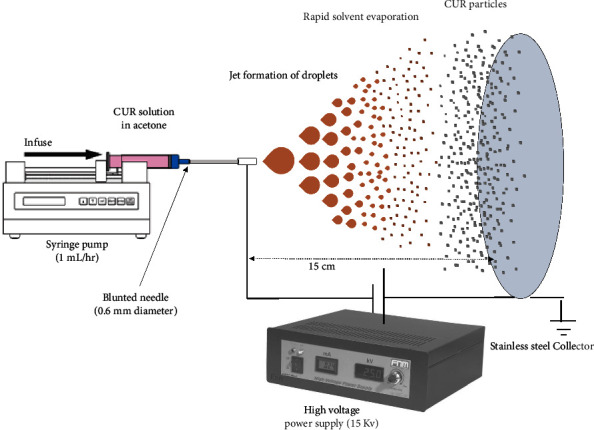
Schematic view of electrospray drying setup.

The ED setup consisted of a syringe pump, metal nozzle, high-voltage power source, and metal collector (Figure 1). We used a single nozzle ED system including a syringe pump (Fanavaran Nano-Meghyas, Tehran Iran), a stainless steel 23 G blunted needle (0.6 mm inner diameter), an FNM high-voltage power supply (Fanavaran Nano-Meghyas, Tehran Iran), and a grounded metal collector plate. The applied parameters were: feed concentrations of 2 and 4% w/v curcumin in acetone, flow rate of 1 mL/h, operating voltage of 15 kV, and tip-to-collector distance of 15 cm, and ambient temperature/pressure. After the ED experiment, the metal plate was put in a vacuum chamber for 24 h to eliminate possible residual acetone. The powder was harvested from the collector and kept in a desiccator at ambient temperature until further analysis.

### 2.3. Production Yield

After each ED experiment, the collected powder from the collector was weighed, and the production yield was obtained by employing the following formula:

(1) $$\begin{array}{c} \text{Production yield}=\frac{\text{weight of collected curcumin powder}}{\text{weight of dissolved curcumin}}\times 100. \end{array}$$

### 2.4. Bulk and Tapped Density

A one milliliter glass syringe was used to determine densities of powder samples, as previously described [34]. First, the initial volume of a certain weight of each powder was measured as the bulk volume by pouring the sample into a plugged 100 unit glass insulin syringe (Becton, Dickinson and Company, USA). Then, the syringe containing the sample was tapped several times on a wooden surface until the powder sample maintained a constant volume and the final volume was recorded as the tapped volume. The bulk and tapped densities were calculated from the ratio of the weights of powder samples and their bulk volume and tapped volume, respectively. The Hausner ratio was calculated by dividing the tapped density by the bulk one. Results are the mean ± standard deviation of the three measurements.

### 2.5. Scanning Electron Microscopy (SEM)

The size and morphology of DPIs and RCUR were assessed using ZEISS Sigma VP field emission scanning electron microscope (ZEISS, Germany). A small amount of powder samples was fixed on aluminum stubs using double-sided carbon tape. Before imaging, the electrical conductivity of particles was enhanced by coating with a gold layer in a desktop sputter coater (DST1 model, Daypetronic co, Tehran, Iran). The images were captured at an acceleration voltage of 10 kV and a 6–8.1 mm working distance. The ImageJ software (1.41v, US National Institutes of Health, USA) was utilized to analyze the particle size distribution of SEM images [35]. For this purpose, the diameters were determined for approximately 160–260 particles from images of different areas. The obtained data were analyzed by GraphPad prism software based on nonlinear curve fitting with the Gaussian as the peak function. To evaluate the uniformity of the obtained particle size distribution, the polydispersity index (PDI) was calculated by the following equation:

(2) $$\begin{array}{c} \text{Polydispersity index}\left(\text{PDI}\right)={\left(\frac{\text{standard deviation}}{\text{mean diameter}}\right)}^{2}. \end{array}$$

### 2.6. Fourier-Transform Infrared Spectroscopy (FTIR)

Chemical structures of RCUR and DPIs were studied by an FTIR (Perkin Elmer, MA, USA). About 2 mg of each powder was added to 200 mg of dry potassium bromide and the mixture compressed into a transparent disc. The disc was then transferred to FTIR analyzer and the spectrum of sample was recorded at the scanning range of 4000 to 450 cm^−1^, resolution of 4 cm^−1^, and scan numbers of 32.

### 2.7. Powder X-Ray Diffraction

The crystalline properties of powder samples were evaluated by Bruker D8 advance X-ray diffractometer (Bruker, Karlsruhe, Germany) operating at ambient conditions, voltage of 40 kV, and current of 40 mA. Aliquots of powder samples were placed on a glass slide, and their XRD spectra were measured between 10 and 45 2*θ* degrees with a step size of 0.02° at a scan rate of 2°/min. The calculated pattern of curcumin Form 1 was generated by applying Mercury software and single crystal structure database.

### 2.8. Differential Scanning Calorimetry (DSC)

A DSC 1 (Mettler-Toledo, Switzerland) was used to evaluate the thermal characteristics of RCUR, EDC2 and EDC4. About 2 mg of the samples were placed in an aluminum pan, crimped using a lid, and then their thermograms were recorded at a scan rate of 10°C/min from 40 to 230°C under nitrogen gas flow. The analysis of thermal data was performed using STARe software (Mettler Toledo, Switzerland).

### 2.9. In Vitro Dissolution Studies

The dissolution rates of 10 mg samples of RCUR and DPIs were investigated by a USP apparatus 2 dissolution tester (Pharma Test co, Germany) in 900 mL of 0.25% SLS at 37.5 ± 0.5°C for up to 120 min at a rotational speed of 100 rpm. At predefined time points of 5, 10, 15, 20, 30, 45, 60, 90, and 120 min, 5 mL samples were collected with replacement with 5 mL of fresh medium, filtered by a syringe filter (pore size 0.22 *μ*m), and analyzed for curcumin concentrations by using a UV spectroscopy (Cecil, UK) at 430 nm. Dissolution efficiency up to 120 min (DE120 min) and amount of drug dissolved in the first 30 min (Q30 min) of samples were calculated by DDsolver program [36]. Data represent the mean ± standard deviation of the three measurements.

### 2.10. In Vitro Deposition Studies by Next-Generation Impactor (NGI)

The in vitro aerodynamic performance of powder samples from a DPI device was studied by an NGI (COPLEY scientific, Nottingham, United Kingdom) equipped with a USP throat and preseparator. Before each run, the NGI cups were covered with Tween 80 1% w/v in ethanol and air-dried to minimize powder bouncing. An amount of 10 ± 1 mg samples was manually filled in size 3 hydroxypropyl methylcellulose capsules (Pure Capsules, DR T&T Health UK Ltd., Corby, UK). The prepared sample was loaded in the holder of an Aerolizer® inhaler device (Novartis, Switzerland), locked, punched, and subsequently connected to the mouthpiece of the NGI. The powders were released into the NGI at a flow rate of 100 L/min for 2.4 s. At this operating condition, the effective aerodynamic cutoff diameter (*μ*m) for NGI stages were 6.12, 3.42, 2.18, 1.31,0.72, 0.40, and 0.24 *μ*m for stages 1 through 7, respectively [30]. One capsule was used for each run, and each powder was tested in triplicate repetitions.

After each actuation, the capsule and inhaler device, throat, preseparator, and stage 1 to the micro-orifice collector (MOC) were washed with known volumes of ethanol to extract and dissolve deposited curcumin powder. The concentrations of curcumin at collected solutions were quantified by UV spectroscopy at 430 nm. To calculate aerosol performance and aerodynamic particle size parameters, the obtained concentration data were processed using Copley inhaler testing data analysis software (COPLEY scientific, Nottingham, United Kingdom).

The total recovered dose (RD) was defined as the amount of curcumin collected from the capsule and device to the MOC of the NGI. The recovered dose percentage (RD%) was calculated as the RD expressed as percentage of the amount of curcumin filled into the capsule. The emitted dose (ED) was defined as the amount of curcumin collected from the throat to the MOC of the NGI. The emitted dose percentage (ED%) was calculated as the ED expressed as the percentage of the RD. The fine particle fraction (FPF) was described as the percentage of the ED having aerodynamic diameter below 5 *μ*m. The Mass median aerodynamic diameter (MMAD) is defined as the diameter at which 50% of the drug mass is collected in larger particles and the remaining 50% is collected within smaller particles. The geometric standard deviation (GSD) is a measure of the spread of an aerodynamic particle size distribution.

### 2.11. Statistical Analysis

All data represent the mean ± standard deviation. The mean and standard deviation (SD) were calculated using Microsoft Excel. Statistical analyses were undertaken in GraphPad Prism software (Version 8.0.2; GraphPad Software, San Diego, CA, USA) by one-way analysis of variance (ANOVA) followed by the post hoc Tukey's test at a significance level of *p*=0.05.

## 3. Results and Discussion

In the present study, we have employed ED to produce pure nanosized DPIs of curcumin with enhanced dissolution and aerosolization profiles and evaluated physicochemical characteristics of produced particles. For ED experiments, acetone was selected as the solvent because it is volatile and has favorable electrical conductivity, low viscosity, and good solvency for curcumin [37]. The feed concentrations of curcumin were chosen to be 2% and 4% w/v based on our recent reports on ED of other drugs for pulmonary delivery [21, 29]. In the next step, the parameters of ED were adjusted to reach a stable jet of the sprayed solution, which is needed for appropriate particle engineering. In the following sections, first, the solid-state properties of the prepared powders are presented and discussed. Then, the dissolution and aerosolization profiles of them are reported and the factors that seem to affect the obtained profiles are discussed.

### 3.1. Production Yield

Under optimized ED condition, the production yields of EDC2 and EDC4 were 53.7 ± 1.1% and 57.4 ± 2.8%, respectively. The obtained yields are comparable to the yield (>50%) that has been considered economically feasible for a lab-scale spray dryer [38]. The disposition of particles at the collection chamber surfaces rather than metal collector and difficulties in removing formed particles from metal collector were responsible for most of the material lost. We worked with a nozzle to collector distance of 15 cm to ensure complete solvent evaporation and prevent particle consolidation in the collector surface. However, previous studies have reported that such long distances lead to reduced yields [39, 40]. To work at shorter distance, the chamber temperature should be elevated or pressure should be lowered to increase the solvent evaporation rate. Therefore, the process yield can be improved by adjusting working distance and modifying the design of the collection chamber and collector [41].

### 3.2. SEM

**Figure 2:**
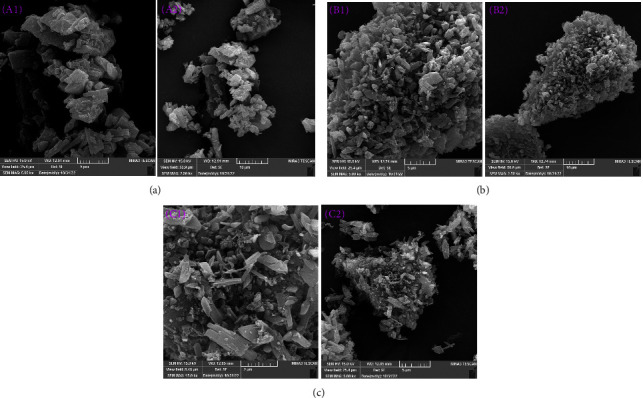
SEM images of RCUR (a), EDC4 (b), and EDC2 (c).

The morphology and particle size of the ED powders in comparison to the RCUR were examined by SEM (Figure 2). Raw curcumin (RCUR) was in an irregular shape, rough in surface textures, and had a broad particle size distribution (Figure 2(a)). Most of the RCUR particles fell in 5–50 *μ*m range with a number of fine crystals adhered to the surfaces of coarse ones. The SEM results revealed that the particle engineering by ED significantly changed the shape and size of curcumin crystals (Figures 2(b) and 2(c)). The ED produced submicron-sized particles with needle- and block-shaped crystals that accumulated to produce foam-like clusters with a rough and porous surface structure in micrometer sizes. The voids within the agglomerates are evident in the SEM images, predicting low densities for ED powders [42]. The observed agglomeration behavior can be explained by the increased free surface energy of nanocrystals [43]. Nanosized particles form groups of larger clusters by weak van der Waals forces to reduce surface energy, and minimize total energy [44]. There are commercialized carrier-free DPIs that have been produced by formulating very fine particles of drug molecules as small-sized soft agglomerates that can deagglomerate during inhalation [45].

**Figure 3:**
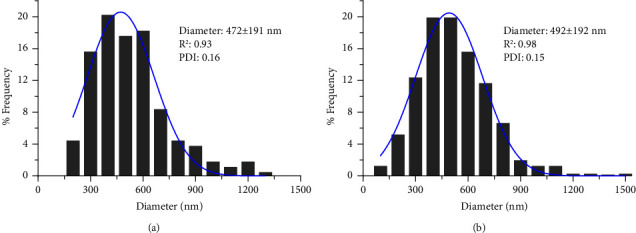
Histograms for particle size distribution, average ± standard deviation, and poly dispersity index (PDI) of the EDC2 (a) and EDC4 (b).

The calculated mean particle sizes for EDC2 and EDC4 were 472 and 492 nm, respectively (Figure 3). It can be seen that increasing concentration from 2 to 4% resulted in a small increase and the size of the formed agglomerated was larger for EDC4. Also, EDC2 seems to contain more needle-shaped and elongated particles. PDI indicates the degree of uniformity of a particle size distribution. PDI values less than 0.3 indicate a homogenous and narrow size distribution of nanoparticles [46]. The PDI values for EDC2 and EDC4 were 0.16 and 0.15, respectively, indicating a narrow unimodal size distribution. A narrow size distribution is essential for dose reproducibility and reducing used dose by targeting particles to deep lung regions and minimizing dose loss in other parts [47].

### 3.3. Powder X-Ray Diffraction

**Figure 4:**
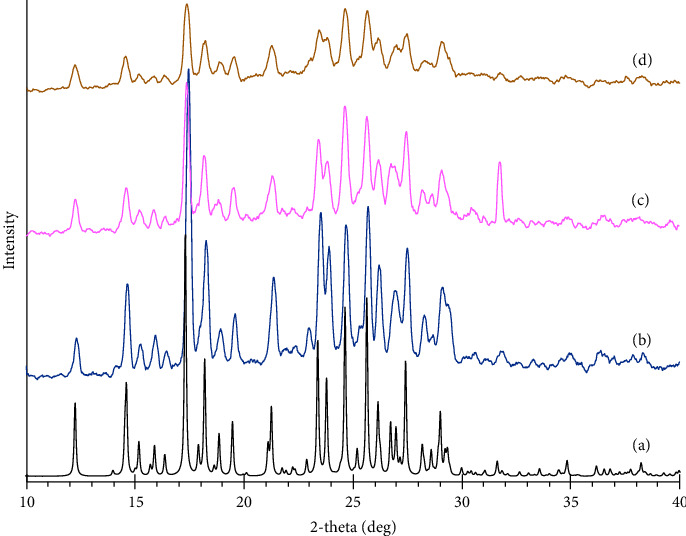
Powder X-ray diffraction patterns of CUR form 1 (a), RCUR (b), EDC2 (c), and EDC4 (d).

Figure 4 represents the XRD patterns of RCUR, EDC2, and EDC4. The pattern of RCUR (Figure 4(b)) displayed characteristic high-intensity diffraction peaks at 2*θ* values of 12.3, 14.6, 15.9, 17.4, 18.3°, 19.8, 19.6, 21.4, 23.5, 23.9, 24.7, 25.7, 26.2, 27.5, and 29.1°, proving its crystalline form and in agreement with reported peaks [48]. Powder X-ray diffraction patterns of EDC2 and EDC4 are shown in Figures 4(c) and 4(d), respectively. For both of these powders, the main diffraction peaks were located at the same set of 2*θ* values as of RCUR, which can be interpreted as preserving crystalline structure after ED. However, reduced peak intensities of engineered powders compared to RCUR, was possibly due to the decreased particle size and crystallinity of these samples [12], which was consistent with the SEM results. The reduction in crystallinity was more pronounced for EDC4 than for EDC2. This behavior can be attributed to differences in the concentration of curcumin in the drying droplets, which led to different crystallization rates [49].

Processing-induced phase transformations during particle engineering methods are very problematic [50]. A well-known type of phase transformation is crystalline to amorphous conversion. Crystalline materials have better physical and chemical stability than amorphous forms [51]. In addition, amorphous forms usually adsorb more moisture than crystalline counterparts, resulting in higher particle agglomeration for amorphous forms and lower depositions in deep lung regions [52]. Another possible challenge for particle engineering techniques is polymorphic changes [53]. CUR has three polymorphic forms [54]. Form 1 is the most stable form and its calculated pattern is shown in Figure 4(a). The PXRD patterns of RCUR, EDC2, and EDC4 matched with the Form 1 pattern. Considering these, it can be concluded that ED produced crystalline particles without inducing polymorphic changes or amorphous phase formation.

### 3.4. FT-IR

**Figure 5:**
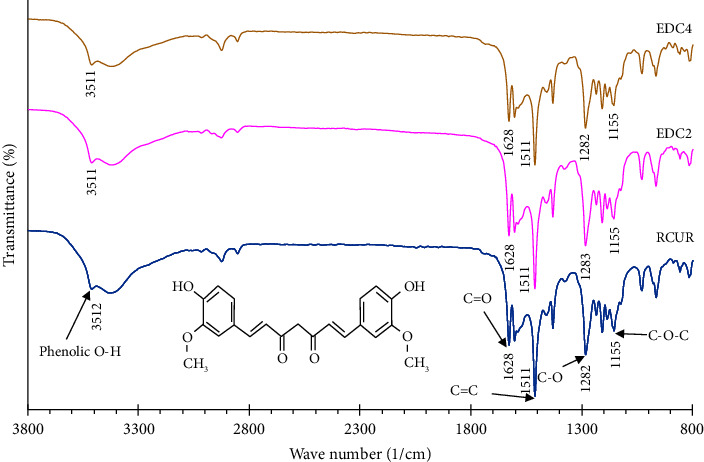
FTIR spectra of studied powder samples.

FTIR was applied to evaluate potential chemical modifications or solid form transformation in the curcumin structure caused by the ED process by comparing the spectra of EDC2 and EDC4 with RCUR (Figure 5). In the spectrum of RCUR, sharp peaks are observed at 1282, 1511, and 1628 cm^−1^ corresponded with the ether group, aromatic carbon group, and carbonyl group, respectively. The peaks of the hydroxyl group were observed at 3300–3500 cm^−1^ regions and 3512 cm^−1^ [55]. As Figure 5 shows, spectra of ED products were similar to RCUR regarding peak shape, intensity, and position, supporting that ED did not affect the chemical integrity of curcumin. Sanphui and coworkers have reported FTIR spectra for the different polymorphs and the amorphous form of CUR [54]. The spectra for RCUR, EDC2, and EDC4 matched with the spectrum of Form 1 in agreement with the obtained PXRD results.

### 3.5. DSC

**Figure 6:**
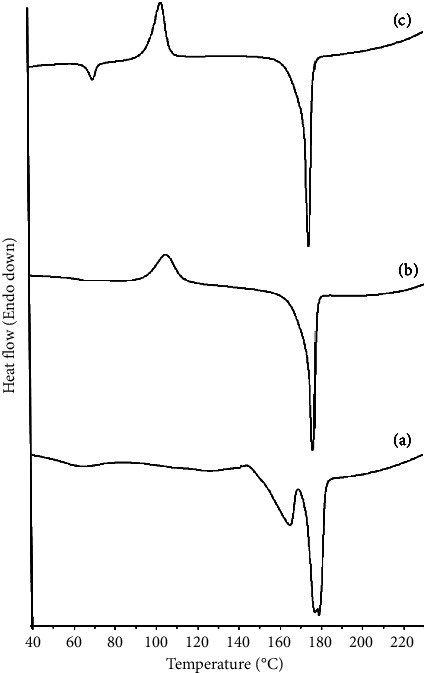
DSC thermograms of RCUR (a), EDC2 (b), EDC4 (c).

DSC Thermograms were recorded to determine the melting points of the studied samples and to investigate occurrence of phase transformations such as polymorphic changes and amorphous phase formation. In the thermogram of RCUR (Figure 6(a)), the melting point endotherm was observed around 180°C, consistent with curcumin Form 1 [54]. Another small endothermic around 165°C in the thermogram of RCUR was possibly due to the presence of other curcuminoids as impurities of RCUR [56]. The thermograms of EDC2 and EDC4 had sharp endotherms around 180°C, showing their crystalline structure as Form 1 (Figures 6(b) and 6(c)). However, their melting endothermic peaks were preceded by the exothermic events around 105°C, suggesting the presence of low quantities of an amorphous phase in these samples [54]. Interestingly, the exothermic peak was more prominent for EDC4, as indicative of its less crystalline structure. These DSC results were in agreement with the PXRD results.

### 3.6. Bulk and Tapped Density

Bulk density, tapped density, and HR of RCUR, EDC2, and EDC4 are presented in Table 1. The densities of studied powders differed considerably with the following rank order: EDC2< EDC4< RCUR. The lower densities of the ED powders can be explained by the presence of porous agglomerates formed by nanosized crystals, as observed by SEM [34]. Powders with a bulk density below 0.3 g/mL and a tapped density below 0.4 g/mL are considered suitable for inhalation drug delivery [57, 58]. Both EDC2 and EDC4 fulfilled these criteria and can achieve a high respirable fraction.

The HR is a qualitative parameter to predict the powder flow. A powder will flow freely in HR less than 1.25, whereas a HR above 1.5 shows poor flowability [59]. The HR of 2.99 indicates extremely poor flowability of RCUR. Our results showed that although ED could reduce HR and improve flowability but could not produce a free-flowing powder (Table 1). The findings also indicated that as the feed concentration of ED was enhanced from 2 to 4%, the HR increased slightly from 1.92 to 2.14 and the flowability decreased. As SEM results showed, particles of EDC2 and EDC4 were in a size range between 100 and 1200 nm. Particles of this low nanometer size exhibit a high surface free energy and are thus very cohesive, showing agglomeration tendency and poor flowability [60]. Such poor flowability has been reported for other carrier-free DPIs such as spray dried ketoprofen [61] and nano-in micro meloxicam [62]. One way to improve flow properties is adding L-leucine or magnesium stearate [63, 64]. For example, adding leucine into the sildenafil carrier-free DPIs improved powder flow and aerodynamic performance [65].

### 3.7. In Vitro Dissolution Study

A major obstacle to the clinical use of curcumin is its low solubility and slow dissolution rate in body fluids. Before it can start its therapeutic effects, curcumin should dissolve in lung lining fluid and permeate through the epithelial layer into lung tissue. He et al. [66] reported that improving the dissolution of curcumin led to higher pulmonary absorption. Therefore, one of the purposes of the current work was to improve dissolution rate of curcumin by particle size reduction to nanometer sizes.

**Figure 7:**
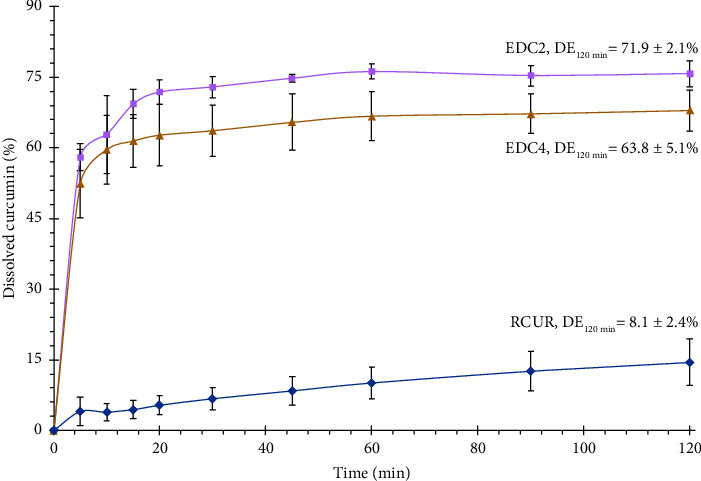
In vitro dissolution profile and DE_120 min_ of RCUR, EDC2, and EDC4 (*n* = 3, mean ± SD).

Figure 7 presents the dissolution profiles of RCUR, EDC2, and EDC4 in the 0.25% SLS dissolution medium. We selected 0.25% SLS as the dissolution medium based on a previous work that investigated the dissolution properties of curcumin nanocrystals prepared by wet milling combined with spray drying for pulmonary delivery [12]. RCUR dissolved very slowly with only 4 and 14% dissolved amount after 15 and 120 min, respectively. On the contrary, EDC2 and EDC4 demonstrated a rapid dissolution with more than 60% dissolved curcumin within the first 15 min. The order of dissolution rates was RCUR < EDC4 < EDC2. The quantitative assessment of dissolution data was conducted by calculating dissolution efficiency up to 120 min (DE_120_). The DE_120_ of EDC2 and EDC4 were 9- and 8-fold higher than RCUR, respectively.

The proposed mechanisms for improving pulmonary absorption by nanocrystal technology are higher solubility and dissolution rate, enhanced mucoadhesion, and enhanced membrane permeability [67]. The dissolution rate of a pure crystal is mainly controlled by its solubility and surface area [68]. For nanocrystals smaller than 100 nm, saturation solubility is enhanced exponentially by decreasing particle size [25]. Therefore, it can be predicted that EDC2 and EDC4 with mean sizes about 500 nm could not significantly enhance solubility of CUR. On the other hand, large surface areas of ED products led to the observed improvements in the dissolution of CUR. The higher dissolution rate of EDC2 compared to EDC4 can be explained by its lower particle size and lower tendency for agglomeration, as explained in the SEM section. These observations were in agreement with previous reports, indicating that particle engineering of water-insoluble drugs as nanocrystals with higher surface area could result in a considerable enhancement in dissolution rates [69].

### 3.8. In Vitro Deposition Profile

**Figure 8:**
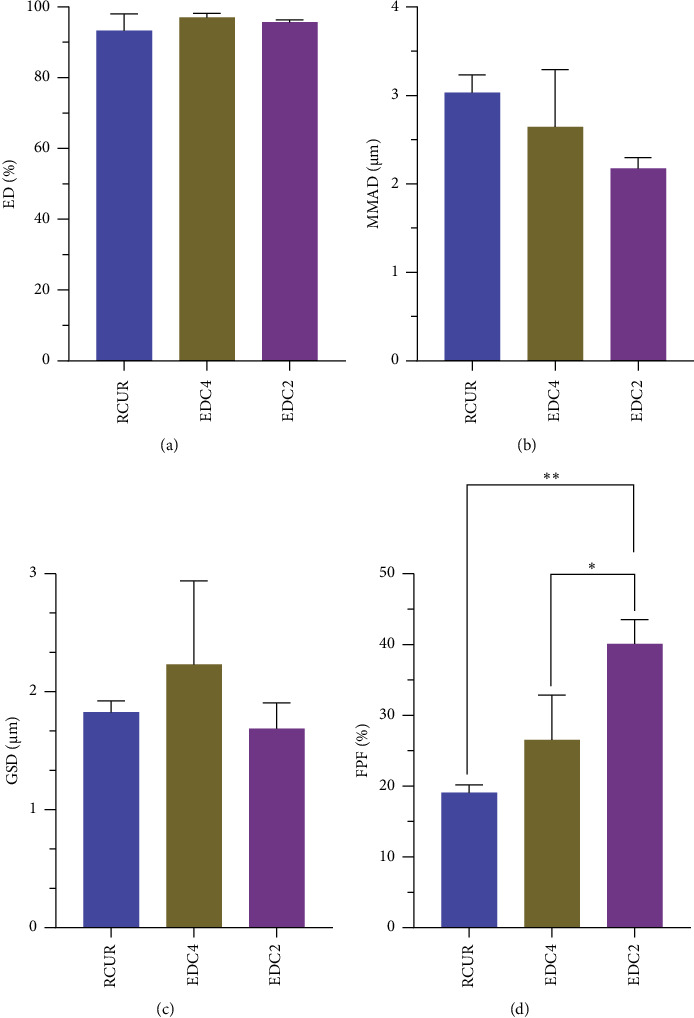
In vitro deposition parameters of studied powders including ED% (a), MMAD (b), GSD (c), and FPF (d) (*n* = 3, ^∗^*p* < 0.05, ^∗∗^*p* < 0.01).

**Figure 9:**
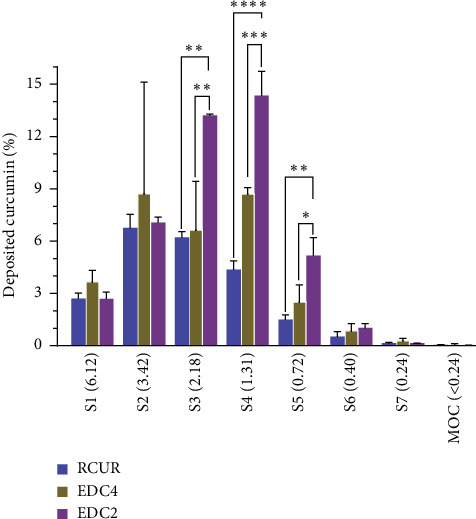
Deposition profiles of CUR, EDC2, and EDC4 at the NGI stages. The aerodynamic cutoff diameter (*μ*m) of each stage is included in parentheses. Samples were fired into NGI at the air flow rate of 100 L/min for 2.4 s employing an Aerolizer® inhaler device (*n* = 3, ^∗^*p* < 0.05, ^∗∗^*p* < 0.01, ^∗∗∗^*p* < 0.001, and ^∗∗∗∗^*p* < 0.0001).

The in vitro aerosolization profiles of the powders were assessed using the NGI (Figures 8 and 9). Recovered dose percentage (RD%), emitted dose percent (ED%) fine particle fraction (FPF), mass median aerodynamic diameter (MMAD), and geometric standard deviation (GSD) were chosen for evaluating the impact of particle engineering on the performance of the curcumin. The RD% between 75 and 125% verifies the accuracy and reproducibility of the method adopted for the collection and analysis of the drug during deposition experiments [70]. In our experiments on RCUR, EDC2, and EDC4, we achieved satisfactory RDs% of 108.5 ± 4.4.5, 113.3 ± 9.7, and 108.3 ± 5.6%, respectively. As Figure 8(a) shows, all studied samples had a high ED% of more than 90% and there was no significant difference between them. These high values indicate that the flow rate of 100 L/min for 2.4 s led to nearly complete release of powders from capsule and device. An important drawback of DPIs formulated as excipient-free aggregates is their high variability (RSD 15%) in the emitted dose [15]. In the current study, we obtained high ED% values (>90%) with very low variability (RSD < 2%) that were above the reported levels (ED% >60) for some commercialized DPIs [71].

Figure 9 presents the data for the percent of curcumin deposited in each stage of NGI for the studied samples. Regardless of the powder type, over 50% of the RDs were primarily deposited in the throat and preseparator of NGI (data not shown). Also, the deposition patterns were approximately similar in stages 1, 2, 6, 7, and MOC. On the other hand, EDC2 exhibited significantly higher depositions at stages 3, 4, and 5 compared to RCUR and EDC4, indicating better deposition performance of EDC2.

Curcumin needs to be mainly delivered to the alveolar region of the lung where it can exhibit therapeutic effects on lung diseases such as chronic obstructive pulmonary disease, pulmonary fibrosis, and cancers [2]. The DPIs with MMADs in the range of 3–10 *μ*m mainly reach nonrespiratory bronchioles, while MMADs ≤3 *μ*m target respiratory bronchioles and alveolar regions [72]. In the current work, the MMADs of EDC2 and EDC4 were less than 3 *μ*m with values of 2.18 ± 0.12 and 2.64 ± 0.65, respectively (Figure 8(b)).

The particle size data of EDC2 and EDC4 (Figure 3) differ significantly from calculated MMAD values by deposition experiments. In addition to particle size, MMAD is affected by other properties, including shape, surface roughness, porosity, density, aggregation, and deagglomeration [42]. The observed differences can be explained by the aggregation tendency of prepared nanocrystals to form micron-sized agglomerates. When the agglomerates are inhaled and interact with lung lining fluid, they can release the curcumin nanocrystals in the lung fluid [66]. The formation of larger agglomerates by fine particles has been reported for other excipient-free DPIs [16, 45]. For example, Hu and coworkers reported that spherical agglomerates of sodium cromoglicate nanoparticles with a diameter of 100 nm size had an MMAD of 4.46 *μ*m [42].

The GSD represents the degree of dispersity in aerodynamic particle size distribution, the lower the GSD number the homogenous the size distribution. The GSD values in the range of 1.5–2.5 *μ*m are considered desirable for DPIs [73]. As Figure 8(c) shows, the lowest GSD was observed for EDC2, reflecting its more homogenous size distribution.

The rank order of FPF achieved by different powders was RCUR < EDC4 < EDC2 (Figure 8(d)). EDC2 represented statistically higher FPF than RCUR (*p* < 0.05) and EDC4 (*p* < 0.01) with about 2-fold increase as compared to RCUR. The higher FPF of EDC2 can be explained by the lower MMAD, lower density, and higher depositions at stages 3–5. Another possible reason is the presence of elongated or needle-like particles within EDC2 (Figure 1). Previous studies have reported that changing particle morphology from block to elongated shape leads to higher FPFs [74, 75]. On the other hand, the same ED% values and different FPF values for EDC2 and RCUR show that RCUR particles could be released from inhaler but could not be dispersed for inhalation. Thus, acceptable RD%, very high ED%, optimum MMAD, small GSD, and high FPF of EDC2 favor delivery of curcumin to the alveolar region.

## 4. Conclusion

In the current study, pure curcumin nanocrystals were successfully prepared by using ED in a single step, at a mild condition, and without need of surfactants. The production yield of the process was acceptable and solid-state analyses showed that formed particle were in a stable crystalline state. Our experiments showed that the feed concentration affects shape, size, aggregation tendency, and crystallinity of the produced particles. It seems that lower feed concentrations lead to better in vitro dissolution and aerodynamic performance. The prepared nanocrystals had a mean particle size of about 500 nm with a narrow unimodal size distribution. These nanosized particles adhered to each other to form micrometer-sized agglomerates. The bulk and tapped density of prepared agglomerates were in the range appropriate for pulmonary delivery; however, their flowability needs to be improved by employing appropriate strategies. Formed nanocrystals could significantly improve the dissolution rate of water-insoluble curcumin. The in vitro aerosolization performance assays demonstrated the suitability of the optimized carrier-free powder for local delivery of curcumin to the lung. The optimized formulation exhibited acceptable RD%, high ED%, optimum MMAD, small GSD, and high FPF that favors the delivery of curcumin to the deep lung regions. The prepared DPI could expand the current delivery options for clinical applications of curcumin in pulmonary diseases. Further studies are required to investigate in vivo effectiveness of the prepared DPI in animal models of asthma and its pulmonary pharmacokinetic profile. Furthermore, the effectiveness of ED method should be proved in pulmonary delivery of other water-insoluble pharmaceutical crystalline agents.

## Figures and Tables

**Table 1:** Bulk density, tapped density, Hausner ratio, and particle morphology of RCUR, EDC2, and EDC4.

Sample	Bulk density (g/mL)	Tapped density (g/mL)	Hausner ratio	Particulate morphology
CUR	0.30 ± 0.01	0.68 ± 0.0.04	2.99 ± 0.14	Plate shaped
EDC2	0.13 ± 0.02	0.25 ± 0.04	1.92 ± 0.07	Needle shaped and block shaped
EDC4	0.16 ± 0.01	0.34 ± 0.01	2.14 ± 0.13	Needle shaped and block shaped

## Data Availability

The data presented in this article are based on the results of the Pharm D thesis of Zahra Hemmati, registered in the School of Pharmacy, Urmia University of Medical Sciences, Urmia, Iran. All data used to support the findings of this study are included within the article.
